# The Diverse Roles of TIMP-3: Insights into Degenerative Diseases of the Senescent Retina and Brain

**DOI:** 10.3390/cells9010039

**Published:** 2019-12-21

**Authors:** Jennifer M. Dewing, Roxana O. Carare, Andrew J. Lotery, J. Arjuna Ratnayaka

**Affiliations:** 1 Clinical and Experimental Sciences, Faculty of Medicine, University of Southampton, MP806, Tremona Road, Southampton SO16 6YD, UK; jmd2g08@soton.ac.uk (J.M.D.); R.O.Carare@soton.ac.uk (R.O.C.); A.J.Lotery@soton.ac.uk (A.J.L.); 2 Eye Unit, University Hospital Southampton NHS Foundation Trust, Southampton SO16 6YD, UK

**Keywords:** TIMP-3, ECM, AMD, sorsby, retina, Alzheimer’s disease, dementia

## Abstract

Tissue inhibitor of metalloproteinase-3 (TIMP-3) is a component of the extracellular environment, where it mediates diverse processes including matrix regulation/turnover, inflammation and angiogenesis. Rare *TIMP-3* risk alleles and mutations are directly linked with retinopathies such as age-related macular degeneration (AMD) and Sorsby fundus dystrophy, and potentially, through indirect mechanisms, with Alzheimer’s disease. Insights into TIMP-3 activities may be gleaned from studying Sorsby-linked mutations. However, recent findings do not fully support the prevailing hypothesis that a gain of function through the dimerisation of mutated TIMP-3 is responsible for retinopathy. Findings from Alzheimer’s patients suggest a hitherto poorly studied relationship between TIMP-3 and the Alzheimer’s-linked amyloid-beta (Aβ) proteins that warrant further scrutiny. This may also have implications for understanding AMD as aged/diseased retinae contain high levels of Aβ. Findings from *TIMP-3* knockout and mutant knock-in mice have not led to new treatments, particularly as the latter does not satisfactorily recapitulate the Sorsby phenotype. However, recent advances in stem cell and in vitro approaches offer novel insights into understanding TIMP-3 pathology in the retina-brain axis, which has so far not been collectively examined. We propose that TIMP-3 activities could extend beyond its hitherto supposed functions to cause age-related changes and disease in these organs.

## 1. Introduction 

Tissue inhibitors of metalloproteinases (TIMPs) are proteins expressed ubiquitously in the body which play important roles through their ability to reversibly inhibit enzymes belonging to the zinc protease superfamily, predominantly matrix metalloproteinases (MMPs) and a disintegrin and metalloproteases (ADAMs) [1]. The TIMP family consists of four members: TIMP-1, -2, -3 and -4. Whilst TIMPs are widely regarded as broad-range MMP inhibitors, each protein exhibits differences in their specificity. TIMP-3, the focus of this review, is found in chromosome 22q12.3 and is nested within an intron of the gene synapsin 3 (*SYN3*); a feature shared with TIMP-1 and TIMP-4 which are located within introns of synapsin 1 (*SYN1*) and synapsin 2 (*SYN2*), respectively [1]. TIMP-2 however, is not associated with any of the synapsin genes. The relationship between synapsin and TIMPs appears to be evolutionarily conserved, having been identified in the fruit fly (*Drosophila melanogaster*) and the tiger blowfish (*Fugu rubripes*) [2], although its nature is yet to be elucidated. There is also considerable conservation in the amino acid structure of TIMP family members. Each protein contains an *N*-terminal domain of approximately 125 amino acids and a *C*-terminal domain of 64 amino acids, with each domain stabilised by three disulphide bonds that form between conserved cysteine residues [1,3]. TIMP-3 is considered to have a predominantly extracellular role, as the protein is capable of binding to the extracellular matrix (ECM) via its *N* or *C*- terminal domains [4,5]. By contrast, TIMP-1, -2 and -4 proteins were considered to predominantly exist in soluble form within the interstitial space of the ECM. However, it has since been shown that these proteins can also interact with cell surface proteins including CD63 and β-1 integrin [6,7,8]. The interaction of TIMP-3 with ECM proteoglycans is primarily mediated via its C-terminal domain. However, binding to the glycosaminoglycan heparin appears to be mediated via the *N*-terminus [9]. Of the four members of the TIMP family, TIMP-3 possesses the broadest range of inhibition, targeting all members of the MMP family and several of the ADAM and ADAMTs (a disintegrin and metalloproteases with thrombospondin motifs) family members. Independent of its inhibitory capabilities, TIMP-3 is also involved in promoting cell proliferation and regulating angiogenesis and apoptosis [1]. Here, experts from the fields of retinal disease and neuropathology have come together to consider the role of TIMP-3, whose myriad roles in diseases of the senescent retina and brain have not yet been examined collectively. We discuss new evidence from our respective laboratories as well as other groups, showing that TIMP-3 activities influence important processes such as tissue remodelling, amyloid-beta (Aβ) pathology and angiogenesis amongst others, and propose that further study could unravel new insights into complex degenerative diseases that have so far eluded effective treatment. 

## 2. Assembly and Disassembly of ECM Molecules: The Dynamic Environment Outside Cells 

**Figure 1:**
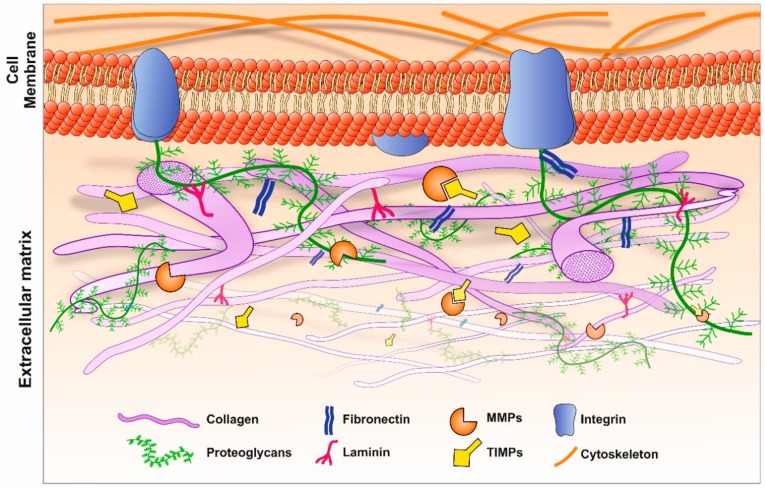
Composition and architecture of the extracellular matrix (ECM). Interactions between key ECM macromolecules including collagens, proteoglycans, laminin and fibronectin facilitates cellular activities such as maintaining homeostasis, differentiation and morphogenesis. ECM turnover is mediated by matrix metalloproteinases (MMPs), which are in turn inhibited by a family of protease inhibitors referred to as tissue inhibitor of metalloproteinases (TIMPs). Amongst the latter group, TIMP-3 feature prominently in the ECM as it binds to the matrix components via its N and C-terminal domains. Cells communicate with ECM components via transmembrane adhesion proteins called integrins which are expressed on their surfaces. A delicate balance between MMPs vs. TIMPs alongside other regulators are required for a healthy ECM.

The ECM is an acellular, protein-rich scaffold in which cells and tissues are embedded. The ECM not only provides structural support but also a dynamic environment in which biochemical and biomechanical cues directly regulate cellular activities such as homeostasis, cell differentiation and morphogenesis [1]. Insights into complex diseases including retinopathies and Alzheimer’s disease (AD) can therefore be found by studying the ECM. The composition of the ECM is relatively heterogeneous; a key characteristic given the range of different tissues and organs it supports, with the ECM configuration ultimately determining the physical and biological properties of resident cells. The two main classes of macromolecules that constitute the ECM are proteoglycans and fibrous proteins. Proteoglycans are composed of long disaccharide chains called glycosaminoglycans (GAGs), covalently bonded to a central core protein. These complex and high molecular weight structures pervade the interstitial space of the ECM to form a gel-like composition, offering a unique buffering, hydration and binding environment that is resistant to mechanical force [10]. The main fibrous proteins of the ECM include collagens, fibronectins, tenascin, elastins and laminin. Collectively, these diverse groups of structural molecules facilitate an ECM capable of numerous complex functions [11]. Of the fibrous constituents of the ECM, the most abundant is collagen, comprising up to 30% of total protein mass and forming the principal structural component [10]. Its primary role is to facilitate tensile strength and biomechanical regulation through cellular adhesion, migration and chemotaxis. There are over 30 known collagen types, although not all are associated with the ECM. In general, a heterogeneous mix of collagen types are observed in the ECM, as in the case of the ocular Bruch’s membrane (BrM), in which collagen type 1, 3–6 are found [12,13]. The BrM supports the overlying retinal pigment epithelium (RPE) which demarcates the blood retinal barrier. However, in a given tissue, a single collagen type may predominate [10]. For instance, type IV forms the principal collagen in cerebral blood vessels, whilst type I collagen constitutes the main variety in bone [14,15]. Collagen production is mostly regulated by interstitial fibroblasts; however, endothelial and epithelial cells have also been shown to be involved in this process [10]. Whilst a subset of cell types may constitutively regulate the synthesis and secretion of collagens for homeostatic purposes, other cells are geared towards responding to external cues. For instance, macrophages have been shown to secrete collagen type VIII in response to atherosclerotic plaques [16,17]. Other ECM components include thrombospondins, cystatins and SPARC (secreted protein acidic and rich in cysteine), which are secreted by constituent cells, which regulate their surrounding environment [18,19,20,21]. SPARC levels in RPE cells were shown to be elevated under ocular pathologies such as proliferative vitreoretinopathy (PVR). Our previous work showed that SPARC was processed through the ER/Golgi pathway and secreted via the RPE basolateral membrane, providing insights into its ECM activities [22]. We also demonstrated that the variant B form of cystatin C, associated with developing age-related macular disease (AMD) was mis-trafficked from the secretory pathway, linking retinopathy with biochemical changes to an ECM constituent [23,24]. Direct interaction between ECM components and the surrounding cells is mediated via cell surface receptors including integrins, discoid domain receptors (DDRs) and proteoglycans [16]. The signals transmitted to cells from the ECM are relayed via chemokines, cytokines and growth factors that are embedded within the ECM and released to interact with receptors. Consequently, a process such as ECM disassembly does not solely serve to detach cells and remove matrix barriers during remodelling, but also releases sequestered signalling molecules by acting as a molecular repository [11] (Figure 1). 

The process of ECM disassembly associated with remodelling and disease is largely mediated by MMPs and ADAMS. There are 23 different MMPs in humans, which can be categorised into six groups, depending on their target substrate as well as sequence and domain homology; collagenases, gelatinases, stromelysin, metrilysins, membrane type MMPs (MT-MMPs) and other MMPs [16,25]. MMPs are translated as an inactive zymogen, referred to as pro-MMPs, the majority of which subsequently become activated in the extracellular space following proteolytic cleavage by serine proteases or other MMPs. Of note, MMPs are only active at low levels under normal homeostatic conditions. However, during development, following injury or during chronic inflammation, their activity increases to enable remodelling and repair of the ECM [10,26]. Gene regulation of MMPs is achieved by transcription factors including hormones and cytokines, as well as mechanical cues such as interactions of cells with the ECM or neighbouring cells [27]. ADAMs are protease enzymes related to MMPs, consisting of membrane-bound ADAMs and secreted ADAMTs [16]. Of the 22 ADAM genes in humans, 12 encode active proteins [26]. Whilst ADAMs primarily act as ‘sheddases’ which cleave transmembrane proteins to release their ectodomain (i.e., cytokines, growth factors and receptors), ADAM10, ADAM12 and ADAM15 are also capable of cleaving ECM proteins [26]. Members of the ADAMTs subfamily have pro-collagen activity which processes and deposits collagen into the ECM. The activities of MMPs and ADAMs are directly regulated by TIMPs, which bind to and inhibit the proteolytic ability of these proteases [1]. 

In recent years, the emerging role of TIMP-3 in processes including inflammation, ECM modulation and cleavage of the amyloid precursor protein (APP) has substantiated the need to better understand its role in complex degenerative diseases such as macular dystrophies and Alzheimer’s disease; a focus of study in our laboratories. TIMP proteins interact directly with the active site of MMPs by non-covalently binding and forming 1:1 stoichiometric ratio complexes [28]. It is important that the expression of TIMP and MMP proteins be tightly regulated to maintain the fine balance between ECM synthesis and degradation, which is critical for healthy tissues. In cardiomyopathy, imbalances in this relationship results in excessive ECM breakdown and collagen loss due to MMP-1 overexpression [29]. Conversely, a significant number of diseases are caused by fibrosis, whereby ECM production exceeds its degradation due to migration and proliferation of ECM-depositing fibroblasts and myofibroblasts. This type of an imbalance results in conditions such as pulmonary fibrosis and cirrhosis [26,30,31]. Other factors that play a role in ECM regulation include TGF-β, which remodel fibroblasts via the SMAD2–SMAD3 transcription complex and promote expression of ECM-related genes such as *COL1A1* and *TIMP-3*, whilst suppressing the expression of the ECM-disassembling enzyme MMP-1 [28]. Disruption of the timely assembly and disassembly of ECM in the aging retina and brain can have significant consequence linked with blindness and dementia. For example, mutations in collagen are implicated in ocular abnormalities such as Knobloch’s and Alport syndrome [32], whilst TIMP-3 mutations are directly linked with Sorsby fundus dystrophy (SFD) [33]. In the brain, MMPs are associated with the degradation of Aβ plaques and inflammation as well as the regulation of cytokines and permeability of the blood brain barrier. Furthermore, expression of MMPs are increased in neurodegenerative disorders including AD [34]. Similarly, collagen VI expression levels are higher in the brains of AD mice models and in human AD patients [35]. Recent discoveries in cell biology and advances in modelling studies, alongside the development of new imaging tools, present new opportunities to consider these dynamic processes and the role of TIMP-3 in a new light. 

## 3. TIMP-3 and Other ECM Changes in the Aging Retina

As early damage to the RPE is thought to play a key role in retinopathy, considerable efforts have been made to study ECM remodelling in response to age and disease in the underlying BrM. The RPE monolayer carries out many functions including the proteolytic degradation of photoreceptor outer segments (POS) from overlying photoreceptors and the recycling of components of the visual cycle as well as the transport of nutrients/metabolites to and from the outer retina. The RPE layer is supported by the acellular pentalaminar BrM, which is strategically positioned between this important tissue and the extraocular environment [12,13,36]. The BrM is a highly organised structure consisting of the basal lamina of the RPE, the inner collagenous layer (ICL), the elastin layer (EL), the outer collagen layer (OCL) and the basal lamina of the choriocapillary endothelium. Thus, the BrM forms both the substratum of the RPE cell layer at one interface as well as the vessel wall (i.e., outer extent) of the choriocapillaris on the other [12]. The structural composition of the BrM is designed to withstand mechanical alterations caused by changes in intraocular pressure and choroidal blood volume to which it stretches and reacts accordingly [12]. There is a linear increase in the thickness of BrM with age, beginning in the retinal periphery, which increases from 2 µm at birth to almost 5 µm by the tenth decade of life [37]. BrM thickening is observed in both the ICL and the OCL, although the latter thickens more prominently. Over time, BrM in the peripheral retina becomes thicker compared to BrM in the central retina (the macula) [12,38,39], indicative of a fundamental imbalance in ECM assembly and disassembly with age. This is evidenced by an increase in collagen cross-linking with age, resulting in a dense collagen matrix that potentially limits accessibility to regulators such as MMPs and reducing collagen turnover in the BrM. Ultimately, this leads to changes in the BrM elasticity and its hydraulic permeability [13]. Furthermore, penetrability of water molecules across the BrM exponentially decreases with age, with the greatest rate of decrease observed in the macula compared to the retinal periphery. Interestingly, the greatest loss of permeability is observed earlier in life, prior to the accumulation of disease-linked debris in the BrM, implying that structural and molecular changes to the BrM may occur in advance of any obvious pathological changes [40,41,42]. Such debris, which are referred to as drusen, appear as electron-dense extracellular deposits between the RPE basal lamina and the ICL of BrM. These can be identified by colour fundus photography as yellow-white deposits, which are typically 30–300 µm in diameter, that are categorised as hard or soft drusen [12,43]. Hard drusen are smaller, refractile deposits identified by their sharp edges and can be present in small numbers in non-diseased eyes of any age. However, the presence of many hard drusen is a risk factor for AMD [44]. Conversely, soft drusen are typically larger, form more diffuse deposits with less distinct edges and have the propensity to become confluent. Their appearance in the macula is associated with a high risk of developing retinal degeneration [44,45]. Drusen consists primarily of lipids and proteins, including acute phase and C-reactive proteins, as well as components of the complement pathway and their inhibitors. The most abundant of these include TIMP-3, clusterin, vitronectin and serum albumin. Proteins in drusen may be further modified by age-related processes such as oxidative stress [46]. Another drusen constituent is the Alzheimer’s-linked Aβ family of misfolding proteins, which is found in high levels in aged donor eyes with moderate–high levels of drusen and in eyes of AMD patients [47,48]. The constituents of drusen are considered to derive from the RPE, photoreceptors and the choroid (endothelial, fibroblast and smooth muscle cells) as well as from serum. Based on mRNA expression data, a majority of drusen constituents were found to originate from serum (20%) and choroidal cells (32%), respectively [13,49]. Lipids also accumulate in the BrM with age, particularly in the macula. These include phospholipids, triglycerides, fatty acids and free cholesterol [50]. Furthermore, age-related lipoprotein-like particles (LLPs) were reported to accumulate in significant amounts in the BrM. These typically vary in size from 60–100 nm, with LLPs as large as 300 nm observed as a result of LLP fusion [51]. In younger eyes (<40 years), LLPs were found to be localised to the EL and OCL and occupied only a small fraction of the space between fibrils. However, with age, LLPs were shown to occupy an increasing volume of elastin inter-fibrillar space and spread to the ICL, eventually forming a lipid sublayer between the ICL and the RPE basal lamina. The source of LLPs is thought to be predominantly RPE in origin, rather than the choriocapillaris, implied by the formation of the lipid layer only once the EL and ICL are filled with LLPs. This suggests that RPE plasma-derived lipoproteins are released into BrM accumulate beneath the basal lamina due to their inability to pass through the ICL and EL [13,51]. Similarly, the source of LLPs is not believed to be derived from photoreceptor phagocytosis by RPE due to the significant presence of esterified cholesterol within BrM LLPs, which are absent from photoreceptors [52]. 

**Figure 2:**
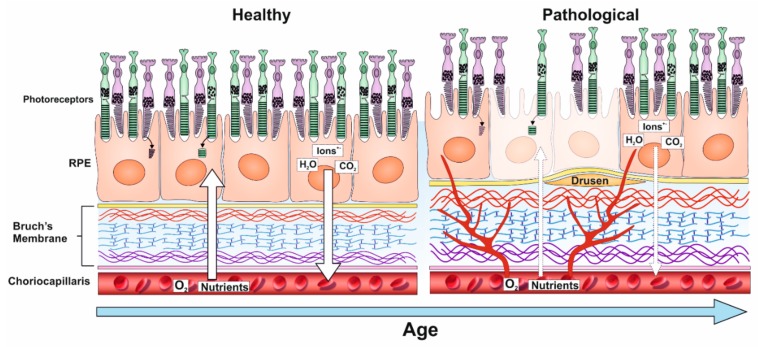
Structural changes to tissues in the outer retina during aging and disease. The retinal pigment epithelium (RPE) carries out activities that are critical to maintaining photoreceptors. The RPE monolayer is supported by a thin tissue called Bruch’s membrane (BrM), which collectively demarcates the blood–retinal barrier. The choriocapillaris provides oxygen and nutrients whilst removing high levels of metabolic waste that are generated in the outer retina. Thickening of the BrM occurs as a natural part of aging. However, changes including the accumulation of sub-RPE protein/lipid deposits referred to as drusen as well as alterations to its biophysical properties are linked with retinopathies such as age-related macular degeneration and Sorsby fundus dystrophy. Late stages of these diseases are associated with the atrophy of RPE cells and blindness due to photoreceptor loss; a phenotype referred to as geographic atrophy. An alternative terminal phenotype, termed choroidal neovascularisation, is characterised by the presence of nascent, leaky capillaries which penetrate the BrM to cause retinal detachment.

Unsurprisingly, BrM pathology is associated with a number of retinal disorders including age-related macular degeneration (AMD), Sorsby fundus dystrophy (SFD), proliferative vitreoretinopathy (PVR), pseudoxanthoma elasticum and Marfan syndrome [12]. It is important to note that the appearance of drusen is not necessarily linked with disease but is rather an indication of normal aging. For instance, 90/100 healthy donors aged between 70 and 80 years were observed to have subclinical macular drusen [13]. Similarly, 65% of drusen proteins were found in drusen from both AMD and healthy patients, with only 33% being exclusive to diseased (AMD) eyes [46]. Consequently, differentiating between normal BrM ageing and BrM alterations linked with pathology can be challenging. However, once normal BrM ageing starts to affect the RPE and photoreceptor function, the transition to pathology may be considered to have begun [13,38]. Considerable efforts have been made in the past to understand BrM changes in retinopathies [53,54]. AMD, in which BrM changes feature prominently, affects ~3% of individuals from mid-life onwards to encompass 1/3 of individuals by the eighth decade of life. Globally, 150 million individuals are affected by early–intermediate stages of AMD, which are largely asymptomatic but include drusen, with a further 10 million individuals suffering from sight-threatening late-stage forms of the disease [55]. Early AMD includes structural and physiological changes to the RPE–BrM complex, which is not always associated with sight loss. BrM pathology is a well-established trigger of AMD, which alongside advanced age, includes increased deposition of complement proteins, hydroxyapatite, modified lipids and Aβ amongst others [45,48,56,57,58]. Alterations to the synthesis and/or activities of TIMP-3 can also be added to this list, as our previous studies identified rare coding *TIMP-3* variants (<0.1%) with a >30-fold increase risk of AMD compared to controls [59]. Patients with rare *TIMP-3* risk alleles (some of which target the coding regions of the TIMP-3 protein) also had a greater association with other AMD risk alleles, suggesting that *TIMP-3* variants contribute to late-onset development of the disease in combination with other susceptibility genes [59,60,61,62]. Collectively, these findings support a model of local, chronic retinal inflammation and ECM changes that lead to well-established markers of pathology, including sub-RPE drusen (basal laminar and linear deposits), in early stages of AMD in at least some patients. These can progress to advanced or late-stage forms, which are classified as either ‘wet’ (choroidal neovascularisation: CNV) or ‘dry’ (geographic atrophy: GA) AMD. CNV is characterised by retinal detachment associated with choroidal angiogenesis; exudates from leaky vessels that compromise the blood-retinal-barrier and formation of fibrotic scar tissue in the macula. By contrast, GA results in a lesion corresponding to areas of photoreceptor and RPE atrophy; the terminal phenotype from aforementioned RPE-BrM early pathology in the macula [63]. Although CNV and GA are reported at similar frequencies [64], patients with GA have no effective treatment. CNV, however, is currently managed in most patients with regular injections of vascular endothelial growth factor (VEGF) inhibitors, although there is evidence to suggest that the disease can switch to a GA phenotype after prolonged treatment in some cases [65] (Figure 2). 

In contrast to complex diseases such as AMD, in which TIMP-3 may play only a contributing factor, some mutations in TIMP-3 are strongly associated with a rare inherited autosomal dominant macula disease called SFD. The similarities between these two retinopathies are such that without genetic screening, those with SFD may be misdiagnosed as AMD patients. SFD is also the only example where a member of the TIMP family appears to be directly linked with a specific disease. SFD causes bilateral loss of central vision due to either RPE atrophy or CNV leading to photoreceptor loss and irreversible blindness. However, disease onset occurs earlier compared to AMD, between the fourth and sixth decade of life but can manifest considerably sooner, with symptoms in patients as young as thirty years old [66]. There are currently 18 known TIMP-3 mutations associated with SFD, with the majority occurring in the last exon of the *TIMP-3* gene (the C-terminus of the protein). Interestingly, many of the mutations result in either a gain or loss of a cysteine amino acid. This is thought to promote the formation of intermolecular disulphide bridges with other mutated TIMP-3 proteins, resulting in TIMP-3 dimers or multimers that are resistant to turnover and clearance from the ageing BrM. Whilst this hypothesis has been supported by several studies [3,67,68,69], others have failed to observe the formation of TIMP-3 dimers [70,71]. For instance, a recent study was unable to confirm the formation of dimers between p.S38C mutant TIMP-3 proteins. However, the study identified evidence for the formation of an aberrant intermolecular disulphide bond between Cys36 andCys38, although it remains unclear as to whether this facilitates a gain or loss of function [71]. The inconsistencies in mutant TIMP-3 dimerisation may be due to differences in the manner in which distinct TIMP-3 mutations affect the protein or due to differences in the way in which these studies were undertaken. An important point to note, however, is that not all TIMP-3 mutations result in a loss or gain of a cysteine, as some mutations generate lysine or arginine residues, or indeed a premature stop codon [68,72,73]. Consequently, it is unclear how the “dimer hypothesis” might account for the wide range of phenotypes reported in SFD patients. It is worthwhile noting that although TIMP-3 is expressed in different tissues, until recently there was no evidence to suggest these mutations caused pathology elsewhere in the body. It was thought that other members of the TIMP family might somehow compensate for any deficiencies of mutated TIMP-3. However, recent findings described two SFD families with a history of pulmonary disease, where individuals over 50 years in one family (carrying the TIMP-3 p.Y191C mutation) were diagnosed with severe emphysema. Similarly, both members of the second family (carrying the TIMP-3 p.S38C mutation) exhibited moderate bronchiectasis [74]. This suggests that some TIMP-3 mutations may be more severe compared to others, or indeed behave very differently than has been previously thought. Furthermore, this highlights the possibility that mutated TIMP-3 proteins could play a role in other systemic syndromes which had hitherto not been considered. The thickening of BrM, a key feature of both SFD and AMD, is thought to increase the relative distance between the choroid and the RPE, and thus potentially limit efficient exchange of nutrients and waste to and from the retina [12]. The lack of vitamin A transport brought about in this manner is thought to underlie night blindness reported by some SFD patients in early disease [33]. Other areas in which mutated TIMP-3 could influence pathology is the manner in which the protein regulates the availability of sequestered ECM signalling molecules to RPE and choroidal endothelial cells that in-turn will influence their behaviour. This, coupled with the different extents to which mutated TIMP-3 could regulate MMPs, is likely to add to the complexity of ocular pathology in SFD and AMD patients. 

## 4. Role of TIMP-3 in the Ageing Brain

AD pathogenesis is driven in part by the presence of misfolding Aβ proteins [75]. The inability to adequately breakdown and/or efficiently remove cerebral Aβ leads to the accumulation of insoluble fibrils in the interstitial fluid that eventually form neurotic plaques [76]. This leads to a cascade of pathological events, including inflammation, synaptic dysfunction and neuronal loss [77]. Under normal physiological conditions, a homeostatic balance between Aβ production and clearance results in low Aβ levels in the brain extracellular fluid [78]. However, this balance is thought to become impaired with age, which increases the risk of developing AD in later life. The APP undergoes proteolytic processing via two alternative and competing mechanisms; the anti-amyloidogenic and the amyloidogenic pathways. The first of these involve cleavage of APP within the Aβ amino acid sequence by α-secretase, leading to the shedding of secreted APP (sAPPα) [77,79]. In the amyloidogenic pathway however, β-secretase cleaves the Aβ sequence at the N terminal, releasing the large APP ectodomain which is further cleaved by γ-secretase, resulting in the formation of Aβ peptides [77]. The amyloid cascade hypothesis has historically occupied the centre-stage in AD [75]. There is, however, growing evidence to show that other factors including metalloproteinases and TIMP-3 also contribute to disease. For instance, ADAM10 and ADAM17 have been identified as APP α-secretases involved in the non-amyloidogenic pathway, with rare variants of these genes associated with AD [80,81,82,83]. TIMP-3 is the only member of the TIMP family capable of inhibiting ADAM10 and ADAM17, which occurs by direct binding to a hydrophobic pocket on the surface of these proteins [84]. ADAM10 and ADAM17 compete with β- and γ-secretases to cleave APP, and consequently through their inhibition, TIMP-3 plays an indirect role in regulating the amyloidogenic pathway and thus Aβ production. This was demonstrated in vitro, where the overexpression of TIMP-3 in neurons increased APP cleavage via β- and γ-secretases resulting in increased Aβ production [85]. Evidence for a link between TIMP-3 and impaired cognition was demonstrated in behavioural tests of *Timp-3* knock-out mice [86]. Increased TIMP-3 levels are also reported in brains of APP transgenic mice as well as in human AD brains where it associates with neurofibrillary tangles (NFTs) and neuritic senile plaques [85,87]. Reduced ADAM10 levels were reported in cerebral spinal fluid of AD patients [88], likely to be correlated with upregulated TIMP-3. However, circulating MMP-9 was also upregulated in plasma from AD patients [89], which is at odds with elevated TIMP-3. It has been proposed that under certain pathological conditions, MMP expression is increased in response to elevated TIMP to promote the inhibitory binding of TIMPs to MMPs, and thus depleting the levels of unbound TIMP available for TIMP receptor-mediated signalling [90]. The mechanisms through which rare risk alleles or mutations in TIMP-3 influence its interactions with ADAM10 and ADAM17 as well as their subsequent cleavage of APP is currently unknown. 

**Figure 3:**
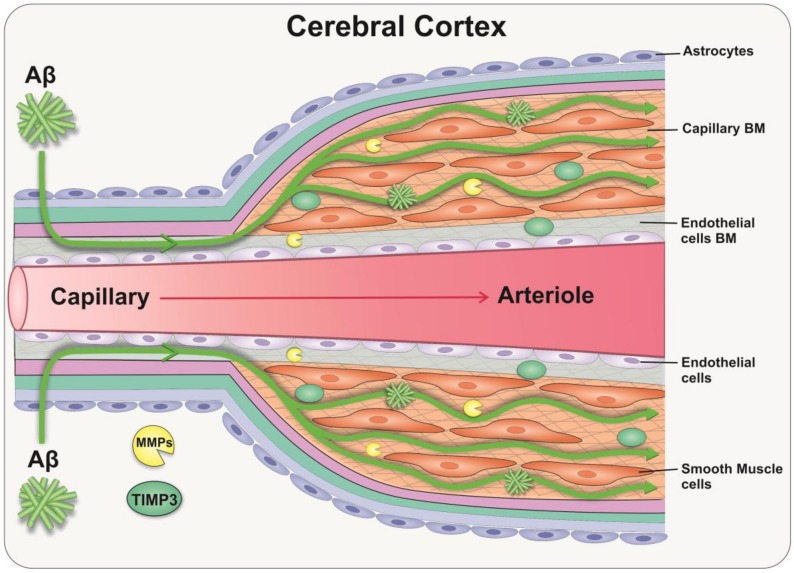
Cross-sectional view of the capillary/arteriole in the cerebral cortex demonstrating clearance of Aβ via the intramural periarterial drainage (IPAD) pathway. Clearance of Aβ from the brain occurs along capillary and periarterial basement membranes and is influenced by the biochemical composition of the vessel’s extracellular matrix (ECM). The age-related changes of cerebral vascular basement membranes favour Aβ deposition and development of cerebral amyloid angiopathy (CAA). Upregulation of TIMP-3 has been observed in Alzheimer’s brains and CAA arteries, suggesting that this ECM regulator may play a key role in regulating IPAD via matrix metalloproteinase (MMP) inhibition.

The accumulation of Aβ in the walls of capillaries and arteries as cerebral amyloid angiopathy (CAA) is a hallmark of AD [91]. In sporadic AD, this accumulation is mainly due to a failure of clearance of interstitial fluid and Aβ from the brain rather than overproduction of Aβ per se [92]. One of the key mechanisms for the elimination of solutes and Aβ from the brain is along the basement membranes of capillaries and arteries, as intramural periarterial drainage (IPAD) [93]. This process depends on the contraction of vascular smooth muscle cells and on the biochemical composition of the ECM that lines the vessel walls [94]. Consistent with aforementioned observations in the retina, changes in the ECM composition with age result in a thickening of the cerebral vascular basement membranes, with an increased ratio of proteoglycans vs. glycoproteins, which favours Aβ aggregation [95]. With these age-related changes of cerebral vessels, the processes driving IPAD fails, favouring the deposition of Aβ and the development of CAA [96,97,98]. Of note, proteomic studies in aged/CAA leptomeningeal arteries demonstrate a significant increase of TIMP-3, suggesting that regulation of the ECM is central to the pathophysiology of Aβ deposition in the cerebral vessels [99]. Cerebral autosomal dominant arteriopathy with subcortical infarcts and leukoencephalopathy (CADASIL) is one of the most common inherited small vessel diseases of the brain, which is characterised by a loss of vascular smooth muscle cells and accumulation of ECM as granular osmiophilic material. The failure to clear these proteins is also central to the development of CADASIL [100]. Consequently, TIMP-3 dysregulation may contribute to the accumulation of ECM proteins and the worsening of vascular pathology in CAA [101]. TIMP-3 may therefore represent an attractive target for therapy in preventing the failure of the IPAD pathway (Figure 3). 

## 5. Role of TIMP-3 in Retinal and Brain Inflammation

The retina and brain are considered immune-privileged sites, offering protection from typical inflammatory responses that occur against foreign and alloantigens in the rest of the body [102]. Nonetheless, chronic inflammatory responses lie at the heart of major diseases such as AMD and AD. Neuroinflammation in the CNS is mediated by cytokines such as tumour necrosis factor-α (TNF-α) that are secreted by microglia and astocytes. ADAM17, also known as TNF-α-converting enzyme (TACE), directly regulate levels of active TNF-α, one of the key players in initiating both local and systemic inflammation. ADAM17 cleaves the membrane-bound pro-TNF-α to its active form, which, once released, triggers a signalling cascade of other cytokines including interleukins and chemokines [4,103]. As TIMP-3 is able to bind and inhibit ADAM17 activity, it could have wide-reaching effects related to TNF-α-mediated inflammation [104]. This is supported by studies in *TIMP-3* knockout mice, which exhibit increased levels of TACE and TNF-α, and develop severe liver inflammation [104,105,106]. However, it is unclear how rare genetic variants and mutations in TIMP-3 may alter the protein’s ability to inhibit TACE and thus limit TNF-α production. It is worthwhile noting that the upregulation of TIMP-3 and its association with NFTs in AD brains may be a compensatory mechanisms aimed at reducing inflammation through the downregulation of TACE [87]. Chronic inflammation may also be driven by mechanisms such as oxidative stress, which feature prominently in ocular pathology. The accumulation of partially digested POS in RPE cells and their modification to pathogenic intracellular aggregates such as lipofuscin is an accepted clinical endpoint in AMD. Such materials emit a distinct spectral signature, which can be quantified as fundus autofluorescence in patients [107,108]. Lipofuscin has been shown to occupy 1% of RPE cytoplasmic volume in the first decade of life, which increases to ~20% by the eighth decade of life [38]. A linear relationship has been established between BrM thickness and RPE autofluorescence, linking these two forms of pathology [109]. Moreover, the accumulation of lipofuscin is also associated with impaired lysosomal activity and triggering of local retinal inflammation, linking deficits in protein clearance with sub-RPE deposits and inflammation [110,111]. Modification to lipofuscin by high levels of retinal photo-oxidation as well as by vitamin A leads to the formation of related intracellular material including pyridinium bis-retinoid A2E, malondialdehyde and 4-hydroxynonenal amongst others [112,113]. Other mechanisms such as the MMP pathway, which is regulated by TIMP-3, has also been shown to contribute to the formation of pathogenic deposits in the ageing retina and brain [54]. The accumulation of lipofuscin in the RPE has been further shown to activate the complement signalling cascade and the secretion of pro-inflammatory cytokines as well as chemokines involved in chronic retinal inflammation [114,115]. Oxidative stress can also directly activate TNF-α and other cytokines including IL-1β, IL-6, and IL-8 [116]. Exposure of RPE cells to these pro-inflammatory cytokines has been shown to downregulate the expression of genes that are critical for normal RPE functioning (*RPE65*, *CDH1*, *RDH5*) whilst upregulating genes (*ZEB1* and *SNAI1*) associated with epithelial to mesenchymal transition (EMT) [117]. The EMT of RPE cells is associated with the development of sub-retinal fibrosis which contribute to sight loss in AMD; further evidence for the role of oxidative stress and inflammation in retinopathies [118,119]. Aβ, which accumulates in aged eyes and in eyes with AMD [48], is another potential source of retinal inflammation, and has been shown to disrupt the RPE barrier via direct and indirect mechanisms, including elevated production of reactive oxygen species, that results in diminished trans-epithelial resistance and barrier integrity [120,121]. How these processes effect the regulation of ECM components such as TIMP-3 in the aged and diseased retina is still not fully understood. 

## 6. TIMP-3 and Angiogenesis in the Retina and Brain 

**Figure 4:**
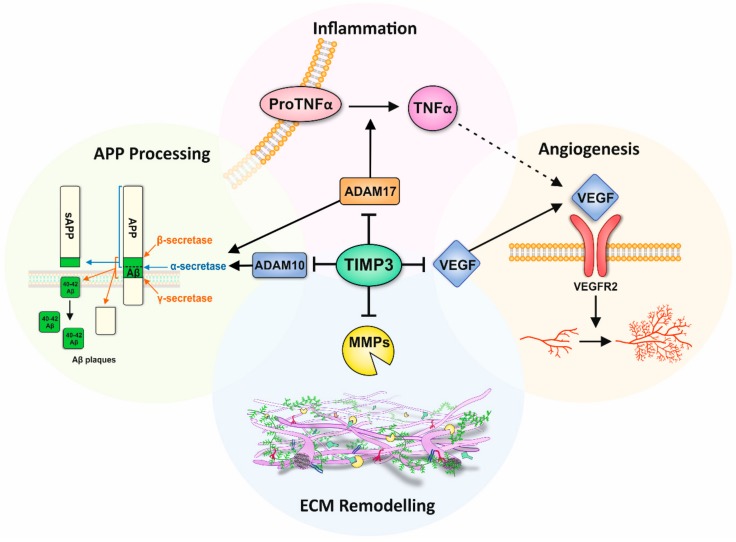
The diverse roles of TIMP-3. TIMP-3 regulates extracellular matrix (ECM) remodelling through inhibition of matrix metalloproteinases (MMP). TIMP-3 is also involved in the processing of the amyloid precursor protein (APP) and Aβ production through inhibition of ADAM10 and ADAM17 (α-secretases). Furthermore, TIMP-3 can inhibit ADAM17 (also referred to as TACE), allowing indirect regulation of TNF-α production form its precursor form. TNF-α is a key mediator of inflammation and angiogenesis though upregulation of the vascular endothelial growth factor (VEGF). However, TIMP-3 can also directly influence angiogenesis, a key determinant of pathology in the late stages of age-related macular degeneration and Sorsby fundus dystrophy, by inhibiting binding of VEGF to its receptor VEGF-R2.

Angiogenesis is described as the process through which nascent blood vessels are formed from pre-existing vessels and is mediated by VEGF and its cognate receptors. Two forms of VEGF receptor have been identified; VEGF-R1 (FLT-1) and VEGF-R2 (KDR). VEGF-R1 has been shown to be crucial in the development of embryonic vasculature whilst VEGF-R2 is involved in endothelial cell development and proliferation [122]. TIMP-3 is capable of competitively inhibiting the binding of VEGF to VEGF-R2, blocking downstream signalling cascades and thus preventing VEGF-mediated angiogenesis. This important TIMP-3 function is believed to be independent of its ability to inhibit MMPs and shown to be mediated by the C-domain of TIMP-3 [122,123]. Angiogenesis involves a multi-step process that requires increased vascular permeability, the disassembly of vessel walls, the degradation of the basement membrane, the migration and invasion of the ECM as well as endothelial cell proliferation [124]. The initial disassembly of the basement membrane and endothelial cell migration is mediated by MMPs. MMP-2 and MMP-9 have been specifically identified as important enzymes required for retinal angiogenesis, such as CNV, which is observed during terminal stages of neovascular AMD [125]. The role of TIMP-3 in inhibiting both VEGF and MMPs suggests that TIMP-3 dysregulation could result in increased CNV. This is supported by studies in *TIMP-3* knockout mice, which exhibit increased MMP activity in the choroid as well as abnormal CNV characterised by irregular choroidal vessels with dilated capillaries. Tissues derived from these animals also show elevated angiogenic activity, which was attenuated in the presence of recombinant TIMP-3 [123]. Furthermore, studies in RPE cells show that TNF-α contributes to CNV via upregulation of VEGF through reactive oxygen species-dependent activation of β-catenin signalling [126]. Our recent work revealed that patients managed for polypoidal choroidal vasculopathy carried the p.S38C TIMP-3 mutation following subsequent genetic testing. [127]. Similarly, we found that another group of patients initially diagnosed with neovascular AMD carried the same p.S38C TIMP-3 mutation [128]. Collectively, our findings support the evidence from other laboratories [122,129] which shows that mutations and/or impairment of TIMP-3 activity causes fundamental changes to the choroid. In AD brains, angiogenesis may also occur as a compensatory mechanism in response to impaired blood flow [130]. VEGF is a key factor in neuropathology, with studies showing that single nucleotide polymorphism in the VEGF promoter region is associated with a risk of AD and vascular dementia [131]. Moreover, the upregulation of the pro-inflammatory cytokine IL-1β has been shown to increase VEGF expression, suggesting a close relationship between inflammation and angiogenesis in AD [132]. In recent years, it has become evident that microglia, the immune cells of the brain, play a key role in the pathogenesis of AD [133]. VEGF and its receptor have been shown to be upregulated in microglia exposed to Aβ. It has been suggested that VEGF and VEGF-R play a role in maintaining the chronic inflammatory response at Aβ plaques and in modulating microglial chemotaxis [134]. Whilst increased VEGF immunoreactivity localised around Aβ plaques have been observed in AD patients, their serum VEGF levels were significantly reduced. However, the latter is associated with significantly increased risk of AD [135,136]. Interestingly, elevated VEGF levels in cerebrospinal fluid were reported to be correlated with cognitive impairment, although another study found no difference between patients and controls [137,138]. Our understanding of the role of TIMP-3 or indeed any effects of rare genetic risk alleles and mutations in TIMP-3 in these processes remain woefully inadequate (Figure 4).

## 7. Concluding Remarks

A number of recent articles, including work from our laboratory, have discussed the biology of TIMP-3 and the pathology caused by mutations in this protein [33,66,71,127,139]. TIMP-3 functions extend beyond the ECM, targeting several other processes, including inflammation as well as angiogenesis in the senescent retina and brain. To our knowledge, the increase in TIMP-3 reported in AD brains has not been reported in other forms of neuropathology, suggesting a specific role for TIMP-3 in AD, possibly through Aβ [85,99]. A question which has arisen frequently is why do mutations in TIMP-3 drive pathology so prominently in the retina, but not in other tissues to the same extent? This was partly answered by an important finding, which showed that individuals carrying the TIMP-3 p.Y191C mutation presented with bronchiectasis and emphysema before developing SFD [74]. This discovery prompted SFD patients attending ophthalmology clinics at Southampton to be screened for any respiratory abnormalities by a specialist. So far, our SFD cohort, which carry the p.S38C and p.S204C mutations, show no evidence of respiratory defects. However, these findings point towards the intriguing possibility that not all TIMP-3 mutations are equal, and that some in fact may result in a more severe phenotype compared to others. This likelihood is also supported by the fact that the age of disease onset varies considerably between SFD patients carrying different TIMP-3 mutations [33]. In contrast to this direct link between TIMP-3 mutations and SFD, the association between TIMP-3 and AMD is less well defined. Our data, which contributed to findings by the International AMD Genomics Consortium, reported a rare (˂0.1%) association between *TIMP-3* and other AMD risk alleles [59]. This suggests that rare genetic variants in *TIMP-3* (which include changes to the *TIMP-3* coding region), alongside other AMD risk alleles, contribute to AMD in a synergistic manner. Intriguingly, why certain *TIMP-3* variants convey increased AMD risk but do not cause SFD is unclear. Perhaps this may be related to the relative location of these rare variants (effect on TIMP-3 protein structure) and/or potential effects in the BrM and choroid (ECM regulation, inflammation etc. that were discussed in this review), which as a whole may be less severe in AMD compared to monogenetic-driven Sorsby pathology. 

In an attempt to understand TIMP-3 biology and the effects of its mutations, investigators have developed several mouse models, with varying degrees of success. *TIMP-3* knockout mice developed irregular choroidal vessels with dilated capillaries and showed increased MMP activity in the choroid. These mice also exhibited increased TACE and TNF-α levels alongside severe liver inflammation [106,140]. However, it is worthwhile noting that, in its absence, other TIMPs may play an ameliorating role, which makes it challenging to delineate a protein-specific pathology. An alternative was to develop a knock-in model, which was created by expressing the TIMP-3 S156C mutation. By 8 months, these mice developed abnormalities in the BrM and RPE, which were evident to some extent in wildtype littermates but only at the advanced age of 30 months. Transgenic mice also showed normal retinal function. Collectively, the pathology observed in *TIMP-3^S156C^* mice supported the current theory of site-specific excess (lack of sufficient TIMP-3 turnover) rather than an absence or deficiency of functional TIMP-3 [69]. Although these models have yielded useful insights, the lack of a robust ocular phenotype consistent with SFD has nevertheless been disappointing. These issues have also cast a shadow on how useful mouse models in general might be for studying TIMP-3 pathology in the brain. The lack of an anatomical macula in rodents as well as potential differences in the ECM turnover rates, immune responses and lifespans between rodents and humans or a combination thereof may account for this. Fortunately, recent advances in molecular biology, imaging technology and in vitro modelling may offer an elegant way forward to study TIMP-3 specific pathology. Induced pluripotent stem cell (iPSC)-RPE from SFD patients cultured for 90 days on transwell inserts spontaneously developed sub-RPE deposits [141]; a hallmark of early pathology. These electron-dense aggregates were significantly numerous in iPSC-RPE cultured from SFD patients compared to those from healthy controls. Furthermore, these deposits, which varied between ≤0.3 and ≥0.51 μm in size, displaced the basolateral RPE membrane and were rich in neutral lipids as well as drusen components ApoE, CryaA/CryaB and TIMP-3. Alongside an upregulation of the complement pathway genes in SFD iPSC-RPE, elevated levels of collagen IV were also observed under pathogenic RPE cells [141]. Consequently, this in vitro model recapitulated several key features of TIMP-3 pathology highlighted in this discussion. The use of cell culture models combined with the use of powerful new imaging techniques can therefore offer the prospect of elucidating TIMP-3 pathology that underlies irreversible sight loss and dementia. Studies of this kind are currently underway in our respective laboratories. 

## References

[1] Brew K., Nagase H. (2010). The tissue inhibitors of metalloproteinases (TIMPs): An ancient family with structural and functional diversity. Biochim. Biophys. Acta.

[2] Solda G., Suyama M., Pelucchi P., Boi S., Guffanti A., Rizzi E., Bork P., Tenchini M.L., Ciccarelli F.D. (2008). Non-random retention of protein-coding overlapping genes in Metazoa. BMC Genom..

[3] Arris C.E., Bevitt D.J., Mohamed J., Li Z., Langton K.P., Barker M.D., Clarke M.P., McKie N. (2003). Expression of mutant and wild-type TIMP3 in primary gingival fibroblasts from Sorsby’s fundus dystrophy patients. Biochim. Biophys. Acta (BBA)—Mol. Basis Dis..

[4] Mohammed F.F., Smookler D.S., Khokha R. (2003). Metalloproteinases, inflammation and rheumatoid arthritis. Ann. Rheum. Dis..

[5] Langton K.P., Barker M.D., McKie N. (1998). Localization of the functional domains of human tissue inhibitor of metalloproteinases-3 and the effects of a Sorsby’s fundus dystrophy mutation. J. Biol. Chem..

[6] Arpino V., Brock M., Gill S.E. (2015). The role of TIMPs in regulation of extracellular matrix proteolysis. Matrix Biol..

[7] Toricelli M., Melo F.H., Peres G.B., Silva D.C., Jasiulionis M.G. (2015). Erratum: Timp1 interacts with beta-1 integrin and CD63 along melanoma genesis and confers anoikis resistance by activating PI3-K signaling pathway independently of Akt phosphorylation. Mol. Cancer.

[8] Jung K.K., Liu X.W., Chirco R., Fridman R., Kim H.R. (2006). Identification of CD63 as a tissue inhibitor of metalloproteinase-1 interacting cell surface protein. EMBO J..

[9] Yu W.H., Yu S., Meng Q., Brew K., Woessner J.F. (2000). TIMP-3 binds to sulfated glycosaminoglycans of the extracellular matrix. J. Biol. Chem..

[10] Frantz C., Stewart K.M., Weaver V.M. (2010). The extracellular matrix at a glance. J. Cell. Sci..

[11] Rozario T., DeSimone D.W. (2010). The extracellular matrix in development and morphogenesis: A dynamic view. Dev. Biol..

[12] Curcio C.A., Johnson M., Ryan S.J. (2013). Structure, Function, and Pathology of Bruch’s Membrane. Retina.

[13] Booij J.C., Baas D.C., Beisekeeva J., Gorgels T.G., Bergen A.A. (2010). The dynamic nature of Bruch’s membrane. Prog. Retin. Eye Res..

[14] Tzaphlidou M. (2008). Bone architecture: Collagen structure and calcium/phosphorus maps. J. Biol. Phys..

[15] Rhodes J.M., Simons M. (2007). The extracellular matrix and blood vessel formation: Not just a scaffold. J. Cell. Mol. Med..

[16] Theocharis A.D., Skandalis S.S., Gialeli C., Karamanos N.K. (2016). Extracellular matrix structure. Adv. Drug Deliv. Rev..

[17] Schnoor M., Cullen P., Lorkowski J., Stolle K., Robenek H., Troyer D., Rauterberg J., Lorkowski S. (2008). Production of Type VI Collagen by Human Macrophages: A New Dimension in Macrophage Functional Heterogeneity. J. Immunol..

[18] Paraoan L., Hiscott P., Gosden C., Grierson I. (2010). Cystatin C in macular and neuronal degenerations: Implications for mechanism(s) of age-related macular degeneration. Vis. Res..

[19] Kay P., Yang Y.C., Hiscott P., Gray D., Maminishkis A., Paraoan L. (2014). Age-related changes of cystatin C expression and polarized secretion by retinal pigment epithelium: Potential age-related macular degeneration links. Investig. Ophthalmol. Vis. Sci..

[20] Howard C., Garcia-Finana M., Yan Q., Hiscott P. (2010). Human retinal pigment epithelial SPARC expression and age: An immunohistochemical study. Histol. Histopathol..

[21] Uno K., Bhutto I.A., McLeod D.S., Merges C., Lutty G.A. (2006). Impaired expression of thrombospondin-1 in eyes with age related macular degeneration. Br. J. Ophthalmol..

[22] Ratnayaka A., Paraoan L., Nelson G., Spiller D.G., White M.R., Hiscott P. (2007). Trafficking of osteonectin by retinal pigment epithelial cells: Evidence for basolateral secretion. Int. J. Biochem. Cell Biol..

[23] Paraoan L., Ratnayaka A., Spiller D.G., Hiscott P., White M.R., Grierson I. (2004). Unexpected intracellular localization of the AMD-associated cystatin C variant. Traffic.

[24] Ratnayaka A., Paraoan L., Spiller D.G., Hiscott P., Nelson G., White M.R., Grierson I. (2007). A dual Golgi- and mitochondria-localised Ala25Ser precursor cystatin C: An additional tool for characterising intracellular mis-localisation leading to increased AMD susceptibility. Exp. Eye Res..

[25] Nagase H., Visse R., Murphy G. (2006). Structure and function of matrix metalloproteinases and TIMPs. Cardiovasc. Res..

[26] Bonnans C., Chou J., Werb Z. (2014). Remodelling the extracellular matrix in development and disease. Nat. Rev. Mol. Cell Biol..

[27] Yamamoto K., Murphy G., Troeberg L. (2015). Extracellular regulation of metalloproteinases. Matrix Biol..

[28] Leivonen S.K., Lazaridis K., Decock J., Chantry A., Edwards D.R., Kahari V.M. (2013). TGF-beta-elicited induction of tissue inhibitor of metalloproteinases (TIMP)-3 expression in fibroblasts involves complex interplay between Smad3, p38alpha, and ERK1/2. PLoS ONE.

[29] Kim H.E., Dalal S.S., Young E., Legato M.J., Weisfeldt M.L., D’Armiento J. (2000). Disruption of the myocardial extracellular matrix leads to cardiac dysfunction. J. Clin. Investig..

[30] Cox T.R., Erler J.T. (2011). Remodeling and homeostasis of the extracellular matrix: Implications for fibrotic diseases and cancer. Dis. Models Mech..

[31] Rauch F., Glorieux F.H. (2004). Osteogenesis imperfecta. Lancet.

[32] Campbell W.A., Deshmukh A., Blum S., Todd L., Mendonca N., Weist J., Zent J., Hoang T.V., Blackshaw S., Leight J. (2019). Matrix-metalloproteinase expression and gelatinase activity in the avian retina and their influence on Müller glia proliferation. Exp. Neurol..

[33] Christensen D.R.G., Brown F.E., Cree A.J., Ratnayaka J.A., Lotery A.J. (2017). Sorsby fundus dystrophy—A review of pathology and disease mechanisms. Exp. Eye Res..

[34] Brkic M., Balusu S., Libert C., Vandenbroucke R.E. (2015). Friends or Foes: Matrix Metalloproteinases and Their Multifaceted Roles in Neurodegenerative Diseases. Mediat. Inflamm..

[35] Cheng J.S., Dubal D.B., Kim D.H., Legleiter J., Cheng I.H., Yu G.-Q., Tesseur I., Wyss-Coray T., Bonaldo P., Mucke L. (2009). Collagen VI protects neurons against Abeta toxicity. Nat. Neurosci..

[36] Benedicto I., Lehmann G.L., Ginsberg M., Nolan D.J., Bareja R., Elemento O., Salfati Z., Alam N.M., Prusky G.T., Llanos P. (2017). Concerted regulation of retinal pigment epithelium basement membrane and barrier function by angiocrine factors. Nat. Commun..

[37] Raan S., Ramrattan R.S., van der Schaft T.L., Mooy C.M., de Bruijn W.C., Mulder P.G.H., de Jong P.T.V.M. (1994). Morphometric analysis of Bruch’s membrane, the choriocapillaris, and the chorioid in aging. Investig. Ophthalmol. Vis. Sci..

[38] Zarbin M.A. (2004). Current concepts in the pathogenesis of age related macular degeneration. Mech. Opthalmic Dis..

[39] Chong N.H.V., Keonin J., Luthert P.J., Frennesson C.I., Weingeist D.M., Wolf R.L., Mullins R.F., Hageman G.S. (2005). Decreased thickness and integrity of the macular elastic layer of Bruch’s membrane correspond to the distribution of lesions associated with age-related macular degeneration. Mol. Pathog. Genet. Inherit. Dis..

[40] Hillenkamp J., Hussain A.A., Jackson T.L., Cunningham J.R., Marshall J. (2004). The Influence of Path Length and Matrix Components on Ageing Characteristics of Transport between the Choroid and the Outer Retina. Investig. Ophthalmol. Vis. Sci..

[41] Starita C., Hussain A.A., Patmore A., Marshall J. (1997). Localization of the site of major resistance to fluid transport in Bruch’s membrane. Investig. Ophthalmol. Vis. Sci..

[42] Moore D.J., Clover G.M. (2001). The effect of age on the macromolecular permeability of human bruch’s membrane. Investig. Ophthalmol. Vis. Sci..

[43] Sarks S., Cherepanoff S., Killingsworth M., Sarks J. (2007). Relationship of Basal laminar deposit and membranous debris to the clinical presentation of early age-related macular degeneration. Investig. Ophthalmol. Vis. Sci..

[44] Bhutto I., Lutty G. (2012). Understanding age-related macular degeneration (AMD): Relationships between the photoreceptor/retinal pigment epithelium/Bruch’s membrane/choriocapillaris complex. Mol. Aspects Med..

[45] Curcio C.A., Johnson M., Rudolf M., Huang J.D. (2011). The oil spill in ageing Bruch membrane. Br. J. Ophthalmol..

[46] Crabb J.W., Miyagi M., Gu X., Shadrach K., West K.A., Sakaguchi H., Kamei M., Hasan A., Yan L., Rayborn M.E. (2002). Drusen proteome analysis. an approach to the etiology of age-related amcular degeneration. Proc. Natl. Acad. Sci. USA.

[47] Anderson D.H., Talaga K.C., Rivest A.J., Barron E., Hageman G.S., Johnson L.V. (2004). Characterization of β amyloid assemblies in drusen: The deposits associated with aging and age-related macular degeneration. Exp. Eye Res..

[48] Lynn S.A., Keeling E., Munday R., Gabha G., Griffiths H., Lotery A.J., Ratnayaka J.A. (2017). The complexities underlying age-related macular degeneration: Could amyloid beta play an important role?. Neural Regen. Res..

[49] Booij J.C., ten Brink J.B., Swagemakers S.M., Verkerk A.J., Essing A.H., van der Spek P.J., Bergen A.A. (2010). A new strategy to identify and annotate human RPE-specific gene expression. PLoS ONE.

[50] Sheraidah G., Steinmetz R., Maguire J., Pauleikhoff D., Marshall J., Bird A.C. (1993). Correlation between Lipids Extracted from Bruch’s Membrane and Age. Ophthalmology.

[51] Huang J.-D., Presley J.B., Chimento M.F., Curcio C.A., Johnson M. (2007). Age-related changes in human macular Bruch’s membrane as seen by quick-freeze/deep-etch. Exp. Eye Res..

[52] Wang L., Li C.M., Rudolf M., Belyaeva O.V., Chung B.H., Messinger J.D., Kedishvili N.Y., Curcio C.A. (2009). Lipoprotein particles of intraocular origin in human Bruch membrane: An unusual lipid profile. Investig. Ophthalmol. Vis. Sci..

[53] Hussain A.A., Lee Y., Zhang J.J., Marshall J. (2011). Disturbed matrix metalloproteinase activity of Bruch’s membrane in age-related macular degeneration. Investig. Ophthalmol. Vis. Sci..

[54] Hussain A.A., Lee Y., Zhang J.J., Francis P.T., Marshall J. (2017). Disturbed Matrix Metalloproteinase Pathway in Both Age-Related Macular Degeneration and Alzheimer’s Disease. J. Neurodegener. Dis..

[55] Wong W.L., Su X., Li X., Cheung C.M.G., Klein R., Cheng C.Y., Wong T.Y. (2014). Global prevalence of age-related macular degeneration and disease burden projection for 2020 and 2040: A systematic review and meta-analysis. Lancet Glob. Health.

[56] Xu Q., Cao S., Rajapakse S., Matsubara J.A. (2018). Understanding AMD by analogy: Systematic review of lipid-related common pathogenic mechanisms in AMD, AD, AS and GN. Lipids Health Dis..

[57] Fernandez-Godino R., Bujakowska K.M., Pierce E.A. (2018). Changes in extracellular matrix cause RPE cells to make basal deposits and activate the alternative complement pathway. Hum. Mol. Genet..

[58] Thompson R.B., Reffatto V., Bundy J.G., Kortvely E., Flinn J.M., Lanzirotti A., Jones E.A., McPhail D.S., Fearn S., Boldt K. (2015). Identification of hydroxyapatite spherules provides new insight into subretinal pigment epithelial deposit formation in the aging eye. Proc. Natl. Acad. Sci. USA.

[59] Fritsche L.G., Igl W., Bailey J.N., Grassmann F., Sengupta S., Bragg-Gresham J.L., Burdon K.P., Hebbring S.J., Wen C., Gorski M. (2016). A large genome-wide association study of age-related macular degeneration highlights contributions of rare and common variants. Nat. Genet..

[60] Abecasis G.R., Yashar B.M., Zhao Y., Ghiasvand N.M., Zareparsi S., Branham K.E., Reddick A.C., Trager E.H., Yoshida S., Bahling J. (2004). Age-related macular degeneration: A high-resolutiongenome scan for susceptibility loci in a population enriched for late-stage disease. Am. J. Hum. Genet..

[61] Lambert N.G., ElShelmani H., Singh M.K., Mansergh F.C., Wride M.A., Padilla M., Keegan D., Hogg R.E., Ambati B.K. (2016). Risk factors and biomarkers of age-related macular degeneration. Prog. Retin. Eye Res..

[62] Chen W., Stambolian D., Edwards A.O., Branham K.E., Othman M., Jakobsdottir J., Tosakulwong N., Pericak-Vance M.A., Campochiaro P.A., Klein M.L. (2010). Genetic variants near TIMP3 and high-density lipoprotein-associated loci influence susceptibility to age-related macular degeneration. Proc. Natl. Acad. Sci. USA.

[63] Kaszubski P., Ben Ami T., Saade C., Smith R.T. (2016). Geographic Atrophy and Choroidal Neovascularization in the Same Eye: A Review. Ophthalmic. Res..

[64] Owen C.G., Jarrar Z., Wormald R., Cook D.G., Fletcher A.E., Rudnicka A.R. (2012). The estimated prevalence and incidence of late stage age related macular degeneration in the UK. Br. J. Ophthalmol..

[65] Lois N., McBain V., Abdelkader E., Scott N.W., Kumari R. (2013). Retinal pigment epithelial atrophy in patients with exudative age-related macular degeneration undergoing anti-vascular endothelial growth factor therapy. Retina.

[66] Anand-Apte B., Chao J.R., Singh R., Stohr H. (2018). Sorsby fundus dystrophy: Insights from the past and looking to the future. J. Neurosci. Res..

[67] Langton K.P., McKie N., Smith B.M., Brown N.J., Barker M.D. (2005). Sorsby’s fundus dystrophy mutations impair turnover of TIMP-3 by retinal pigment epithelial cells. Hum. Mol. Genet..

[68] Langton K.P., McKie N., Curtis A., Goodship J.A., Bond P.M., Barker M.D., Clarke M. (2000). A novel tissue inhibitor of metalloproteinases-3 mutation reveals a common molecular phenotype in Sorsby’s fundus dystrophy. J. Biol. Chem..

[69] Weber B.H., Lin B., White K., Kohler K., Soboleva G., Herterich S., Seeliger M.W., Jaissle G.B., Grimm C., Reme C. (2002). A mouse model for Sorsby fundus dystrophy. Investig. Ophthalmol. Vis. Sci..

[70] Qi J.H., Ebrahem Q., Yeow K., Edwards D.R., Fox P.L., Anand-Apte B. (2002). Expression of Sorsby’s fundus dystrophy mutations in human retinal pigment epithelial cells reduces matrix metalloproteinase inhibition and may promote angiogenesis. J. Biol. Chem..

[71] Naessens S., De Zaeytijd J., Syx D., Vandenbroucke R.E., Smeets F., Van Cauwenbergh C., Leroy B.P., Peelman F., Coppieters F. (2019). The N-terminal p.(Ser38Cys) TIMP3 mutation underlying Sorsby fundus dystrophy is a founder mutation disrupting an intramolecular disulfide bond. Hum. Mutat..

[72] Lin R.J., Blumenkranz M.S., Binkley J., Wu K., Vollrath D.A. (2006). Novel His158Arg Mutation in TIMP3 Causes a Late-Onset Form of Sorsby Fundus Dystrophy. Am. J. Ophthalmol..

[73] Saihan Z., Li Z., Rice J., Rana N.A., Ramsden S., Schlottmann P.G., Jenkins S.A., Blyth C., Black G.C., McKie N. (2009). Clinical and biochemical effects of the E139K missense mutation in the TIMP3 gene, associated with Sorsby fundus dystrophy. Mol. Vis..

[74] Meunier I., Bocquet B., Labesse G., Zeitz C., Defoort-Dhellemmes S., Lacroux A., Mauget-Faysse M., Drumare I., Gamez A.S., Mathieu C. (2016). A new autosomal dominant eye and lung syndrome linked to mutations in TIMP3 gene. Sci. Rep..

[75] Benilova I., Karran E., De S.B. (2012). The toxic Abeta oligomer and Alzheimer’s disease: An emperor in need of clothes. Nat. Neurosci..

[76] Rosenberg G.A. (2009). Matrix metalloproteinases and their multiple roles in neurodegenerative diseases. Lancet Neurol..

[77] Lichtenthaler S.F. (2011). alpha-secretase in Alzheimer’s disease: Molecular identity, regulation and therapeutic potential. J. Neurochem..

[78] Yin K.J., Cirrito J.R., Yan P., Hu X., Xiao Q., Pan X., Bateman R., Song H., Hsu F.F., Turk J. (2006). Matrix metalloproteinases expressed by astrocytes mediate extracellular amyloid-beta peptide catabolism. J. Neurosci..

[79] Chow V.W., Mattson M.P., Wong P.C., Gleichmann M. (2010). An overview of APP processing enzymes and products. Neuromol. Med..

[80] Kim M., Suh J., Romano D., Truong M.H., Mullin K., Hooli B., Norton D., Tesco G., Elliott K., Wagner S.L. (2009). Potential late-onset Alzheimer’s disease-associated mutations in the ADAM10 gene attenuate {alpha}-secretase activity. Hum. Mol. Genet..

[81] Hartl D., May P., Gu W., Mayhaus M., Pichler S., Spaniol C., Glaab E., Bobbili D.R., Antony P., Koegelsberger S. (2018). A rare loss-of-function variant of ADAM17 is associated with late-onset familial Alzheimer disease. Mol. Psychiatry.

[82] Postina R., Schroeder A., Dewachter I., Bohl J., Schmitt U., Kojro E., Prinzen C., Endres K., Hiemke C., Blessing M. (2004). A disintegrin-metalloproteinase prevents amyloid plaque formation and hippocampal defects in an Alzheimer disease mouse model. J. Clin. Investig..

[83] Kuhn P.H., Wang H., Dislich B., Colombo A., Zeitschel U., Ellwart J.W., Kremmer E., Rossner S., Lichtenthaler S.F. (2010). ADAM10 is the physiologically relevant, constitutive alpha-secretase of the amyloid precursor protein in primary neurons. EMBO J..

[84] Gooz M. (2010). ADAM-17: The enzyme that does it all. Crit. Rev. Biochem. Mol. Biol..

[85] Hoe H.S., Cooper M.J., Burns M.P., Lewis P.A., van der Brug M., Chakraborty G., Cartagena C.M., Pak D.T., Cookson M.R., Rebeck G.W. (2007). The metalloprotease inhibitor TIMP-3 regulates amyloid precursor protein and apolipoprotein E receptor proteolysis. J. Neurosci..

[86] Baba Y., Yasuda O., Takemura Y., Ishikawa Y., Ohishi M., Iwanami J., Mogi M., Doe N., Horiuchi M., Maeda N. (2009). Timp-3 deficiency impairs cognitive function in mice. Lab. Investig..

[87] Dunckley T., Beach T.G., Ramsey K.E., Grover A., Mastroeni D., Walker D.G., LaFleur B.J., Coon K.D., Brown K.M., Caselli R. (2006). Gene expression correlates of neurofibrillary tangles in Alzheimer’s disease. Neurobiol. Aging.

[88] Sogorb-Esteve A., Garcia-Ayllon M.S., Gobom J., Alom J., Zetterberg H., Blennow K., Saez-Valero J. (2018). Levels of ADAM10 are reduced in Alzheimer’s disease CSF. J. Neuroinflamm..

[89] Lorenzl S. (2003). Increased plasma levels of matrix metalloproteinase-9 in patients with Alzheimer’s disease. Neurochem. Int..

[90] Moore C.S., Crocker S.J. (2012). An alternate perspective on the roles of TIMPs and MMPs in pathology. Am. J. Pathol..

[91] Attems J. (2005). Sporadic cerebral amyloid angiopathy: Pathology, clinical implications, and possible pathomechanisms. Acta Neuropathol..

[92] Mawuenyega K.G., Sigurdson W., Ovod V., Munsell L., Kasten T., Morris J.C., Yarasheski K.E., Bateman R.J. (2010). Decreased clearance of CNS beta-amyloid in Alzheimer’s disease. Science.

[93] Carare R.O., Bernardes-Silva M., Newman T.A., Page A.M., Nicoll J.A., Perry V.H., Weller R.O. (2008). Solutes, but not cells, drain from the brain parenchyma along basement membranes of capillaries and arteries: Significance for cerebral amyloid angiopathy and neuroimmunology. Neuropathol. Appl. Neurobiol..

[94] Aldea R., Weller R.O., Wilcock D.M., Carare R.O., Richardson G. (2019). Cerebrovascular Smooth Muscle Cells as the Drivers of Intramural Periarterial Drainage of the Brain. Front. Aging Neurosci..

[95] Kalaria R.N. (1997). Cerebrovascular degeneration is related to amyloid-beta protein deposition in Alzheimer’s disease. Ann. N. Y. Acad. Sci..

[96] Hawkes C.A., Jayakody N., Johnston D.A., Bechmann I., Carare R.O. (2014). Failure of perivascular drainage of beta-amyloid in cerebral amyloid angiopathy. Brain Pathol..

[97] Keable A., Fenna K., Yuen H.M., Johnston D.A., Smyth N.R., Smith C., Al-Shahi Salman R., Samarasekera N., Nicoll J.A., Attems J. (2016). Deposition of amyloid beta in the walls of human leptomeningeal arteries in relation to perivascular drainage pathways in cerebral amyloid angiopathy. Biochim. Biophys. Acta.

[98] Hawkes C.A., Gatherer M., Sharp M.M., Dorr A., Yuen H.M., Kalaria R., Weller R.O., Carare R.O. (2013). Regional differences in the morphological and functional effects of aging on cerebral basement membranes and perivascular drainage of amyloid-beta from the mouse brain. Aging Cell.

[99] Manousopoulou A., Gatherer M., Smith C., Nicoll J.A., Woelk C.H., Johnson M., Kalaria R., Attems J., Garbis S.D., Carare R.O. (2016). Systems proteomic analysis reveals that clusterin and tissue inhibitor of metalloproteinases 3 increase in leptomeningeal arteries affected by cerebral amyloid angiopathy. Neuropathol. Appl. Neurobiol..

[100] Carare R.O., Hawkes C.A., Jeffrey M., Kalaria R.N., Weller R.O. (2013). Review: Cerebral amyloid angiopathy, prion angiopathy, CADASIL and the spectrum of protein elimination failure angiopathies (PEFA) in neurodegenerative disease with a focus on therapy. Neuropathol. Appl. Neurobiol..

[101] Monet-Lepretre M., Haddad I., Baron-Menguy C., Fouillot-Panchal M., Riani M., Domenga-Denier V., Dussaule C., Cognat E., Vinh J., Joutel A. (2013). Abnormal recruitment of extracellular matrix proteins by excess Notch3 ECD: A new pathomechanism in CADASIL. Brain.

[102] Forrester J.V., Xu H. (2012). Good news-bad news: The Yin and Yang of immune privilege in the eye. Front. Immunol..

[103] Amour A., Slocombe P.M., Webster A., Butler M., Knight C.G., Smith B.J., Stephens P.E., Shelley C., Hutton M., Knäuper V. (1998). TNF-α converting enzyme (TACE) is inhibited by TIMP-3. FEBS Lett..

[104] Smookler D.S., Mohammed F.F., Kassiri Z., Duncan G.S., Mak T.W., Khokha R. (2006). Cutting Edge: Tissue Inhibitor of Metalloproteinase 3 Regulates TNF-Dependent Systemic Inflammation. J. Immunol..

[105] Kassiri Z., Oudit G.Y., Sanchez O., Dawood F., Mohammed F.F., Nuttall R.K., Edwards D.R., Liu P.P., Backx P.H., Khokha R. (2005). Combination of tumor necrosis factor-alpha ablation and matrix metalloproteinase inhibition prevents heart failure after pressure overload in tissue inhibitor of metalloproteinase-3 knock-out mice. Circ. Res..

[106] Mohammed F.F., Smookler D.S., Taylor S.E., Fingleton B., Kassiri Z., Sanchez O.H., English J.L., Matrisian L.M., Au B., Yeh W.C. (2004). Abnormal TNF activity in Timp3-/- mice leads to chronic hepatic inflammation and failure of liver regeneration. Nat. Genet..

[107] Kauppinen A., Paterno J.J., Blasiak J., Salminen A., Kaarniranta K. (2016). Inflammation and its role in age-related macular degeneration. Cell. Mol. Life Sci..

[108] Holz F.G., Sadda S.R., Staurenghi G., Lindner M., Bird A.C., Blodi B.A., Bottoni F., Chakravarthy U., Chew E.Y., Csaky K. (2017). Imaging Protocols in Clinical Studies in Advanced Age-Related Macular Degeneration: Recommendations from Classification of Atrophy Consensus Meetings. Ophthalmology.

[109] Okubo A., Rosa R.H., Bunce C.V., Alexander R.A., Fan J.T., Bird A.C., Luthert P.J. (1999). The relationships of age changes in retinal pigment epithelium and Bruch’s membrane. Investig. Ophthalmol. Vis. Sci..

[110] Ferrington D.A., Sinha D., Kaarniranta K. (2016). Defects in retinal pigment epithelial cell proteolysis and the pathology associated with age-related macular degeneration. Prog. Retin. Eye Res..

[111] Piippo N., Korhonen E., Hytti M., Kinnunen K., Kaarniranta K., Kauppinen A. (2018). Oxidative Stress is the Principal Contributor to Inflammasome Activation in Retinal Pigment Epithelium Cells with Defunct Proteasomes and Autophagy. Cell. Physiol. Biochem. Int. J. Exp. Cell. Physiol. Biochem. Pharmacol..

[112] Eldred G.E., Lasky M.R. (1993). Retinal age pigments generated by self-assembling lysosomotropic detergents. Nature.

[113] Sparrow J.R., Gregory-Roberts E., Yamamoto K., Blonska A., Ghosh S.K., Ueda K., Zhou J. (2012). The bisretinoids of retinal pigment epithelium. Prog. Retin. Eye Res..

[114] Nociari M.M., Lehmann G.L., Perez Bay A.E., Radu R.A., Jiang Z., Goicochea S., Schreiner R., Warren J.D., Shan J., Adam de Beaumais S. (2014). Beta cyclodextrins bind, stabilize, and remove lipofuscin bisretinoids from retinal pigment epithelium. Proc. Natl. Acad. Sci. USA.

[115] Zhou J., Kim S.R., Westlund B.S., Sparrow J.R. (2009). Complement Activation by Bisretinoid Constituents of RPE Lipofuscin. Investig. Ophthalmol. Vis. Sci..

[116] Hernandez-Zimbron L.F., Zamora-Alvarado R., Ochoa-De la Paz L., Velez-Montoya R., Zenteno E., Gulias-Canizo R., Quiroz-Mercado H., Gonzalez-Salinas R. (2018). Age-Related Macular Degeneration: New Paradigms for Treatment and Management of AMD. Oxid. Med. Cell. Longev..

[117] Kutty R.K., Samuel W., Boyce K., Cherukuri A., Duncan T., Jaworski C., Nagineni C.N., Michael Redmond T.M. (2016). Proinflammatory cytokines decrease the expression of genes critical for RPE function. Mol. Vis..

[118] Kobayashi M., Tokuda K., Kobayashi Y., Yamashiro C., Uchi S.H., Hatano M., Kimura K. (2019). Suppression of Epithelial-Mesenchymal Transition in Retinal Pigment Epithelial Cells by an MRTF-A Inhibitor. Investig. Ophthalmol. Vis. Sci..

[119] Hyttinen J.M.T., Kannan R., Felszeghy S., Niittykoski M., Salminen A., Kaarniranta K. (2019). The Regulation of NFE2L2 (NRF2) Signalling and Epithelial-to-Mesenchymal Transition in Age-Related Macular Degeneration Pathology. Int. J. Mol. Sci..

[120] Cao L., Wang H., Wang F. (2013). Amyloid-beta-induced matrix metalloproteinase-9 secretion is associated with retinal pigment epithelial barrier disruption. Int. J. Mol. Med..

[121] Bruban J., Glotin A.L., Dinet V., Chalour N., Sennlaub F., Jonet L., An N., Faussat A.M., Mascarelli F. (2009). Amyloid-beta(1-42) alters structure and function of retinal pigmented epithelial cells. Aging Cell.

[122] Qi J.H., Ebrahem Q., Moore N., Murphy G., Claesson-Welsh L., Bond M., Baker A., Anand-Apte B. (2003). A novel function for tissue inhibitor of metalloproteinases-3 (TIMP3): Inhibition of angiogenesis by blockage of VEGF binding to VEGF receptor-2. Nat. Med..

[123] Janssen A., Hoellenriegel J., Fogarasi M., Schrewe H., Seeliger M., Tamm E., Ohlmann A., May C.A., Weber B.H., Stohr H. (2008). Abnormal vessel formation in the choroid of mice lacking tissue inhibitor of metalloprotease-3. Investig. Ophthalmol. Vis. Sci..

[124] Hoffmann S., He S., Ehren M., Ryan S.J., Wiedemann P., Hinton D.R. (2006). MMP-2 and MMP-9 secretion by RPE is stimulated by angiogenic molecules found in choroidal neovascular membranes. Retina.

[125] Lambert V., Wielockx B., Munaut C., Galopin C., Jost M., Itoh T., Werb Z., Baker A., Libert C., Krell H. (2003). MMP-2 and MMP-9 synergize in promoting choroidal neovascularization. FASEB J..

[126] Wang H., Han X., Wittchen E.S., Hartnett M.E. (2016). TNF-alpha mediates choroidal neovascularization by upregulating VEGF expression in RPE through ROS-dependent beta-catenin activation. Mol. Vis..

[127] Koutresi D., Clarke B., Lotery A.J., De Salvo G. (2019). Sorsby fundus dystrophy with polypoidal choroidal vasculopathy: Extending TIMP3 phenotypes. Clin. Exp. Ophthalmol..

[128] Warwick A., Gibson J., Sood R., Lotery A. (2016). A rare penetrant TIMP3 mutation confers relatively late onset choroidal neovascularisation which can mimic age-related macular degeneration. Eye Lond.

[129] Qi J.H., Dai G., Luthert P., Chaurasia S., Hollyfield J., Weber B.H., Stohr H., Anand-Apte B. (2009). S156C mutation in tissue inhibitor of metalloproteinases-3 induces increased angiogenesis. J. Biol. Chem..

[130] Jefferies W.A., Price K.A., Biron K.E., Fenninger F., Pfeifer C.G., Dickstein D.L. (2013). Adjusting the compass: New insights into the role of angiogenesis in Alzheimer’s disease. Alzheimers Res. Ther..

[131] Chapuis J., Tian J., Shi J., Bensemain F., Cottel D., Lendon C., Amouyel P., Mann D., Lambert J.C. (2006). Association study of the vascular endothelial growth factor gene with the risk of developing Alzheimer’s disease. Neurobiol. Aging.

[132] Fahey E., Doyle S.L. (2019). IL-1 Family Cytokine Regulation of Vascular Permeability and Angiogenesis. Front. Immunol..

[133] Hansen D.V., Hanson J.E., Sheng M. (2018). Microglia in Alzheimer’s disease. J. Cell Biol..

[134] Ryu J.K., Cho T., Choi H.B., Wang Y.T., McLarnon J.G. (2009). Microglial VEGF Receptor Response Is an Integral Chemotactic Component in Alzheimer’s Disease Pathology. J. Neurosci..

[135] Tang H., Mao X., Xie L., Greenberg D.A., Jin K. (2013). Expression level of vascular endothelial growth factor in hippocampus is associated with cognitive impairment in patients with Alzheimer’s disease. Neurobiol. Aging.

[136] Mateo I., Llorca J., Infante J., Rodríguez-Rodríguez E., Fernández-Viadero C., Pena N., Berciano J., Combarros O. (2007). Low serum VEGF levels are associated with Alzheimer’s disease. Acta Neurol. Scand..

[137] Corsi M.M., Licastro F., Porcellini E., Dogliotti G., Galliera E., Lamont J.L., Innocenzi P.J., Fitzgerald S.P. (2011). Reduced plasma levels of P-selectin and L-selectin in a pilot study from Alzheimer disease: Relationship with neuro-degeneration. Biogerontology.

[138] Chakraborty A., Chatterjee M., Twaalfhoven H., Del Campo Milan M., Teunissen C.E., Scheltens P., Fontijn R.D., van Der Flier W.M., de Vries H.E. (2018). Vascular Endothelial Growth Factor remains unchanged in cerebrospinal fluid of patients with Alzheimer’s disease and vascular dementia. Alzheimer’s Res. Ther..

[139] Dewing J., Christensen D.R.G., Hongisto H., Scott J., Jenkins B., Cree A.J., Skottman H., Ratnayaka J.A., Lotery A. (2019). Modelling Sorsby’s Fundus Dystrophy using patient-derived iPSC-RPE. Investig. Ophthalmol. Vis. Sci..

[140] Kassiri Z., Oudit G.Y., Kandalam V., Awad A., Wang X., Ziou X., Maeda N., Herzenberg A.M., Scholey J.W. (2009). Loss of TIMP3 enhances interstitial nephritis and fibrosis. J. Am. Soc. Nephrol. JASN.

[141] Galloway C.A., Dalvi S., Hung S.S.C., MacDonald L.A., Latchney L.R., Wong R.C.B., Guymer R.H., Mackey D.A., Williams D.S., Chung M.M. (2017). Drusen in patient-derived hiPSC-RPE models of macular dystrophies. Proc. Natl. Acad. Sci. USA.

